# Platelet Rich Plasma in the Treatment of Bisphosphonate-Related Osteonecrosis of the Jaw: Personal Experience and Review of the Literature

**DOI:** 10.1155/2014/298945

**Published:** 2014-06-10

**Authors:** F. Longo, A. Guida, C. Aversa, E. Pavone, G. Di Costanzo, L. Ramaglia, F. Ionna

**Affiliations:** ^1^Division of Maxillofacial & ENT Surgery, Department of Melanoma, Sarcoma and Head and Neck Surgery, Istituto Nazionale Tumori-Fondazione G. Pascale-IRCCS, Via Aniello Falcone 186, 80127 Naples, Italy; ^2^Postgraduate School in Oral Surgery, Department of Neurosciences, Reproductive and Odontostomatological Sciences, University of Naples “Federico II”, Via Pansini 5, 80131 Naples, Italy; ^3^Division of Transfusion Medicine, Department of Haematology, Istituto Nazionale Tumori-Fondazione G. Pascale-IRCCS, Via Semmola 1, 80131 Naples, Italy

## Abstract

Bisphosphonates (BPs) are a class of synthetic drugs commonly used to treat bone metastasis and various bone diseases that cause osseous fragility (such as osteoporosis). Bisphosphonate-related osteonecrosis of the jaw (BRONJ) is a common complication in patients who received BPs, especially intravenously. Recently, osteonecrosis of the jaw (ONJ) caused by chemotherapeutic not belonging to BPs drug class has been reported. For this reason, it has been proposed recently to rename BRONJ in antiresorptive agents related osteonecrosis of the jaw (ARONJ), to include a wider spectrum of drugs that may cause osteonecrosis of the jaw. The most debated topic about ARONJ/BRONJ is therapy. The most adequate procedure is far from being standardized and prevention seems to play a pivotal role. In our study, we considered 72 patients with BRONJ with nonsurgical therapy, surgical therapy, and surgical therapy with platelet rich plasma (PRP) gel to evaluate its therapeutic effect in promoting ONJ wounds healing. Good results showed by PRP in improving wound healing give away to case-control randomized studies that could give definitive evidence of its effectiveness.

## 1. Introduction

Bisphosphonates (BPs) are a class of synthetic drugs commonly used to treat bone metastasis and various bone diseases that cause osseous fragility (such as osteoporosis). They are able to inhibit bone resorption and prevent loss of bone mass with consequent pathologic fractures, pain, and/or hypercalcemia. They can be divided into two major groups, nitrogen-containing and nonnitrogen-containing bisphosphonates, according to the presence or absence of a nitrogen atom located in the R2 group, with two different mechanisms of action on osteoclasts [1, 2].

Bisphosphonate-related osteonecrosis of the jaw (BRONJ) is a pathological condition in which there is presence of exposed necrotic bone in the maxillofacial region lasting for more than 8 weeks in a patient who has received BPs and has not received radiation therapy to craniofacial region [3, 4]. There is also a “nonexposed” variant of BRONJ, where no necrotic bone is exposed, but radiographic abnormality with bone pain and swelling is present [5]. Recently, osteonecrosis of the jaw (ONJ) caused by chemotherapeutic not belonging to BPs drug class agents such as sunitinib (multikinase inhibitors) [6], bevacizumab, and everolimus (monoclonal antibody that targets vascular endothelial growth factor) [7] has been reported in patients who never have taken BPs [8]. For this reason, it has been proposed recently to rename BRONJ in antiresorptive agents related osteonecrosis of the jaw (ARONJ), to include a wider spectrum of drugs that may cause osteonecrosis of the jaw [9].

Pathogenesis of BRONJ is still unclear, but the inhibition of osteoclasts (which leads to impaired natural remodeling process, that is, a critical event for bone healing) and inhibition of angiogenesis (which slows down the healing of bone and soft tissues) are thought to play a key role. BRONJ is usually triggered by local traumas like tooth extractions, other minor dentoalveolar surgeries, and dentures [4, 10–12]. There has been reported spontaneous occurrence too [13] which is commonly caused by underlying odontogenic/periodontal infection. Anyway, it must be said that genetic/individual susceptibility is strongly involved in pathogenesis, since BRONJ does not occur in all patients [14].

Diagnosis of BRONJ is usually performed radiologically (panoramic radiographs, dental cone beam computed tomography, or spiral computed tomography). Osteolysis, osteosclerosis, thickening of lamina dura, thickening of periosteum, widening of periodontal space, subperiosteal bone formation or sequestra, fracture, and radiologic evidence of sinusitis [15] are usually seen in BRONJ lesions. Where clinically nonexposed necrotic bone can be seen, further exams such as bone scintigraphy, PET scans, or MRI may help in identifying early areas of bone involvement [16].

However, these radiological examinations have very poor specificity and similar findings may be caused in other diseases like odontogenic infections, bone involvement in multiple myeloma, or bone primary tumor/metastasis. An accurate anamnesis is thus necessary. The American Association of Oral and Maxillofacial Surgeons (AAOMS) suggested a staging system based on four stages of BRONJ/ARONJ [5, 17] as follows:

- stage zero is represented by the nonexposed variant, where other symptoms and signs as pain, sinus tracts, or radiologic markers are present [18];
- first stage includes asymptomatic bone exposure;
- second and third stage include patients with exposed bone of various extent with other concomitant symptoms and signs which are mainly a result of secondary infection of the necrotic bone. The symptoms may include increased tooth mobility, formation of sinus tracts, suppuration and traumatic ulceration of oral mucosa adjacent to exposed bone, mandibular fracture, or cervical lymphadenopathy [19].

The most debated topic about ARONJ/BRONJ is therapy. The most adequate procedure is far from being standardized and prevention seems to play a pivotal role.

Physicians who intend to treat ARONJ usually have their own protocol, which is, usually, based on drug therapy for low stage ONJs and surgical therapy (curettage or en bloc removal) for advanced stages or resistant cases [20, 21].

In our study, we treated 72 patients with BRONJ with nonsurgical therapy; in nonresponsive cases, surgical therapy or surgical therapy with platelet rich plasma (PRP) gel was performed.

## 2. Materials and Methods

Seventy-two patients affected by BRONJ observed at the Division of Maxillofacial & ENT Surgery, of “Istituto Nazionale Tumori, Fondazione G. Pascale-IRCCS,” Naples, Italy, from May 2006 to August 2013 were included in this study. Their data/tumour history is summarised in Table 1.

All patients were treated with bisphosphonates (alendronate, pamidronate, or zoledronic acid) and developed osteonecrosis of the jaw. The duration of treatment with BPs varied from 4 to 62 months.

The extension and the features of the osteonecrosis were evaluated by clinical examination and radiographically with panoramic X-rays scan and CT scan. According to AAOMS suggestions, the lesions were classified as stage 0 in five cases, stage 1 in eleven, stage 2 in forty-one, and stage 3 in fifteen.

Gender, age, primary disease, and administered drug were retrospectively examined and reported in Table 1.

All patients with every grade (0, 1, 2, or 3) of lesions underwent a two-week nonsurgical treatment (per os 500 mg ciprofloxacin and chlorhexidine 0,20% mouth rinse, twice a day); thus, the status of the lesion(s) was updated. If the lesion had healed, they underwent a regular follow-up; if the lesion had improved, they continued therapy for other two weeks; if the lesion had not improved or worsened, they underwent surgical treatment (curettage or curettage + excision of necrotic bone) or surgical treatment with PRP (curettage or curettage + excision of necrotic bone, placement of autologous PRP in the residual wound, and closure of the wound), continuing the nonsurgical treatment. All the 72 patients thus underwent nonsurgical treatment; unsuccessful nonsurgical patients were therefore moved to the surgical treatment group, for a total of 15 patients treated with surgery only and 34 patients treated with surgery and PRP.

All patients underwent regular follow-up, from 6 to 94 months.

### 2.1. Preparation of PRP

Autologous platelet gel was prepared at the IRCCS Pascale Foundation Transfusion Medicine OU on the same day of application; multiple samples of whole blood (total 60–100 mL) were taken from each patient and collected in 10 mL ACD vacutainers (Becton Dickinson Labware, Franklin Lakes, NJ). The amount of blood taken from each patient was based on the size, extension, and depth of the lesion to be treated. Blood was then centrifuged at 180 rpm per 10′, in order to separate concentrated erythrocytes from platelet rich plasma (PRP). Afterward, PRP was centrifuged for 10′ at 1800 rpm to separate platelet concentrate (PC) from platelet poor plasma (PPP). This process yielded 10 mL of PC, at a final concentration of 1000 × 103/*μ*L roughly, for every 60 mL of blood.

Thrombin, used to activate platelets and accelerate the gelling process, was prepared by adding calcium gluconate to the autologous PPP, at a ratio of 0.2 mL : 1 mL, under a laminar-flow hood (Faster Bio48). After 15–40 minutes of incubation at 37°, to allow for thrombin formation, the product was centrifuged once again at 1800 g for 10–15 minutes. Then, 1 mL autologous thrombin-containing supernatant was added to the previously separated PRP, together with 0.5 mL ionized Ca in a Petri dish (Falcon, Becton Dickinson Labware), which was shaken until a gelatinous mixture was obtained (from 2 to 10 minutes).

With this technique, autologous PLT gel can be prepared in the lab in about 90 minutes; if not used in the same day, it must be aliquoted and stored at −40°C before gelling. Before administration, each sample was checked for sterility (culturing for aerobic and anaerobic bacteria and mycetes) and quality (platelet concentration in PRP).

### 2.2. Statistical Analysis

Different outcomes among groups were analyzed and then their statistical significance was evaluated with chi-square test (significant when *χ* ^2^ < 0.05) and *P* value (significant when *P* < 0.05).

## 3. Results

Of 72 patients, 23 had complete response with nonsurgical treatment only, 15 underwent surgical treatment without PRP (8 with complete response and 7 with partial response), and 34 underwent surgical treatment with PRP (32 with complete response and 2 with partial response), as summarised in Table 2.

Success rate according to stage at diagnosis is summarized in Table 3; if stage 0 (100% of success) was not considered, no statistical difference in outcome has been found among the other staging groups.

Successful therapeutic pathway according to diagnosis stage is summarised in Table 4. For a stage 0 BRONJ, nonsurgical management was successful in every case (100%). Nonsurgical management success rate decreases in subsequent stages (stage I: 72%; stage II: 20%; stage III: 13%).

When analyzing groups of patients who pursued the two surgical pathways (with or without PRP), PRP group was found statistically significantly more successful (*P* = 0.003) than the surgery without PRP group.

Surgery without PRP group has shown low success percentage (53%), much lower than the PRP group (94%). Surgery with PRP group and surgery without PRP group did not show any significant difference in successful outcome among the different stages.

## 4. Discussion and Conclusions

Management of BRONJ is a controversial topic. Clear bone exposure is often complicated by secondary infections of the denuded bone leading to development of osteomyelitis, with abscess or fistula formation and even pathologic fractures may occur [3]. To avoid these events, which have a severe impact on the quality of life of the affected patients, different approaches have been proposed [22].

### 4.1. Nonsurgical Management

This approach includes antibiotics and antifungals (systemic or topical) in addition to disinfectant mouthwashes and appropriate analgesia [21, 23–27].

Some authors recommend that exposed bone should be irrigated with 0.12% chlorhexidine every 72 h for 4 weeks rather than the use of chlorhexidine mouthwash only.

It has been suggested that, before systemic antimicrobials are prescribed, wound or pus samples, or both, should be harvested for microscopy and sensitivity testing, including testing for the presence of* Actinomyces* spp. 1,5.

Among systemic antimicrobials, penicillin-based ones are commonly and widely used (phenoxymethylpenicillin, amoxicillin, co-amoxiclav, or clindamycin with or without metronidazole) [4, 25, 27, 28].

It must be highlighted that the duration of this treatment is not standardized, and suggestions range from between 7 and 15 days to very much longer treatment [27–31].

It may also be applied as a palliative approach in patients with ONJ and aggressive cancers with very poor prognosis, for whom more extensive treatment is not indicated [9].

Many authors report that nonsurgical management treats local infection and stops the progression of BRONJ even if it does not lead to the resolution of all mucosal and osseous lesions, because exposed bone in itself is not a problem [5, 28, 32].

In the short term, a conservative approach has many benefits for those who do not have advanced stage disease. Anyway relapses and progression of the disease are very common events even in patients who respond well initially [33, 34].

### 4.2. Surgical Management

Surgical approach founds its rationale on the evidence that exposed bone, with its sharp/irregular edges and sequestrum formation, amplifies the risk of increasing inflammation and superinfection and thus should be eliminated. Although there is a general consensus on this last topic, it is the extent of surgical intervention that causes the most debate [4, 28, 35]. Deciding the necessary quote of bone that must be removed is indeed the most difficult decision of any surgical approach proposed so far [24]. For example, French guidelines highlight that, as BPs are administered systemically, actually all margins surrounding BRONJ lesions are affected and thus should be resected [35–37]. It is a common procedure to perform resections at least until a margin of “normally bleeding” bone is obtained, as bleeding indicates a metabolic potential for healing.

Using a Wood's lamp after administration of tetracycline (250 mg four times a day for at least 3 days) or doxycycline (100 mg twice daily for 10 days) has also been suggested to help to delineate radical resection margins [38, 39].

Histologic examination of tissues should be performed only when there is a justified suspicion of underlying malignancy, because it causes further stress to soft/osseous tissues, which may exacerbate the condition [29, 30]. Types of surgical managements can be thus classified into local interventions and radical interventions.

### 4.3. Local Intervention

Local intervention is a surgical approach which does not involve operating on the basal bone of the mandible or maxilla, therefore removing loose or developing bony sequestra alone, but not all the necrotic zone en bloc, with minimal disturbance of overlying soft tissues and low risk of consequent bone fracture [13]. It avoids the exposure of further bone, and positive outcomes in at least 80% of cases have been reported [23–25, 29, 40–43].

Guidelines from the British Dental Association (BDA) and the American Society of Bone and Mineral Research (ASBMR) suggest a conservative surgical approach in case of small segments of necrotic bone which have not caused pathological fractures, removing sharp edges to prevent soft tissue trauma [31, 43]. Moreover, antibiotics and mouthwashes are prescribed similarly to the nonsurgical approach.

Many authors suggest the use of local flaps to expose the necrotic bone, thus aiding removal of the necrotic bone and primary closure of the wound [23, 33, 40, 42, 44–46].

Most authors recommend conservative treatment in most patients and then switching to more aggressive surgical protocol in refractory cases [20, 28, 36].

### 4.4. Radical Intervention

In radical management, “marginal resections” (resection of the alveolus without loss of mandibular continuity) and “segmental resections” (mandibular continuity is broken and reconstructed with bone plates) are performed. Large sections of jawbones are taken away, aiming at removing all the necrotic bone and resecting bone beyond the alveolus. AAOMS recommends using this approach in stage 3 BRONJ particularly, when lesions are very large or there is a pathological fracture [31, 43].

Authors who perform radical interventions usually report excellent results in terms of healing. Anyway, this approach exposes a major issue, which is reconstructing the defect. Options include immediate or delayed rigid plate fixation or bone graft; an obturator is recommended for maxillary defects [24, 36].

As patients thus undergo major surgical intervention(s) with this approach, medical indications for surgery must be wisely considered, as BRONJ patients are often debilitated oncologic individuals [22, 36].

### 4.5. Platelet Rich Plasma

Use of PRP has been suggested by many authors to enhance postsurgical wound healing. PRP gel represents a relatively new technique, which seems, thanks to the action of multiple growth factors, to increase tissue vascularization, overtaking one of the major factors on pathogenesis of ONJs, the lack of vascularization. In addition, it is autologous and therefore it is a biocompatible and safe product. The growth factors in PRP promote angiogenesis and bone and mucosal healing. All studies report excellent results, but, as ours, they are neither case controlled nor randomized [22, 47–54].

## 5. Conclusions

Considering what emerges from literature reviewing and our personal experience, we consider it useful to start with any patient at any stage with a two-week nonsurgical approach. Even if it has been successful in low percentage in advanced BRONJ stages, we consider avoiding unnecessary surgical intervention to these patients a priority, avoiding both useless stress and surgical related risks; furthermore, when nonsurgical approach does not succeed, a two-week delay in performing surgery does not expose patients to major risks. Anyway, symptoms referred by patients (especially pain) must always be considered in planning treatment.

Given the necessity of properly suturing wounds when using PRP gel to enable its permanence, patients who might have had difficulties in lugging wound flaps were not included in the PRP group. Possibly for this reason, surgery without PRP group has shown low success percentage (53%), much lower than the PRP group (94%). These data and observation that surgery with PRP group and surgery without PRP group did not show any significant difference in successful outcome among the different stages highlight the importance of a satisfying closure in the complete healing of BRONJ wounds.

Good results showed by PRP in improving wound healing give a way to case-control randomized studies that could give definitive evidence of its effectiveness.

Nowadays, BRONJ management is still a controversial topic, and there is no definitive standard of care for this disease, with prevention playing a fundamental key role [12, 20, 55]. Treatment for lower stages should be conservative as possible. For advanced stages or cases refractory to nonsurgical approach, surgical resection of the necrotic bone [56] should be performed, possibly granting a proper suture of margins and, according to good reported results, inserting PRP in the residual postsurgical wound. In any case, a try of nonsurgical treatment in every patient seems mandatory.

## Figures and Tables

**Table 1:** Patients data.

	Frequency
Gender (PRP)	
Male	12
Female	60
Age at diagnosis	
Minimum	37
Maximum	81
Mean	59
Primary tumor (PRP)	
Prostate	9
Breast	54
Lung	8
Multiple myeloma	1
Bisphosphonate	
Pamidronate	22
Alendronate	2
Zoledronic acid	48
Cause	
Tooth extraction	47
Prosthetic/dental trauma	25
Periodontal disease	15
ONJ status at diagnosis	
Stage 0	5
Stage I	11
Stage II	41
Stage III	15

**Table 2:** Response according to treatment.

Success rates according to treatment	Frequency (%)
Nonsurgical treatment (72)	
Complete response	23 (32%)
Partial response	49 (78%)
Surgical treatment without PRP (15)	
Complete response	8 (53%)
Partial response	7 (47%)
Surgical treatment with PRP (34)	
Complete response	32 (94%)
Partial response	2 (6%)

**Table 3:** Treatment response according to stage at diagnosis.

Success rates according to diagnosis stage	Frequency (%)
Stage 0 (5 patients)	
Complete response	5 (100%)
Partial response	0
Stage I (11 patients)	
Complete response	9 (81%)
Partial response	2 (19%)
Stage II (41 patients)	
Complete response	31 (76%)
Partial response	10 (24%)
Stage III (15 patients)	
Complete response	11 (73%)
Partial response	4 (27%)

**Table 4:** Successful approaches according to stage at diagnosis.

Successful therapeutic pathway according to diagnosis stage	Frequency (%)
Stage 0 (5 patients)	
Nonsurgical	5 (100%)
Surgical without PRP	0
Surgical with PRP	0
Stage I (11 patients)	
Nonsurgical	8 (72%)
Surgical without PRP	2 (18%)
Surgical with PRP	1 (10%)
Stage II (41 patients)	
Nonsurgical	8 (20%)
Surgical without PRP	7 (17%)
Surgical with PRP	26 (63%)
Stage III (15 patients)	
Nonsurgical	2 (13%)
Surgical without PRP	6 (40%)
Surgical with PRP	7 (47%)
